# Modulation of macrophage metabolism as an emerging immunotherapy strategy for cancer

**DOI:** 10.1172/JCI175445

**Published:** 2024-01-16

**Authors:** Corey Dussold, Kaylee Zilinger, Jillyn Turunen, Amy B. Heimberger, Jason Miska

**Affiliations:** Department of Neurological Surgery and Lou and Jean Malnati Brain Tumor Institute at Northwestern Medicine, Robert H. Lurie Comprehensive Cancer Center, Feinberg School of Medicine, Northwestern University, Chicago Illinois, USA.

## Abstract

Immunometabolism is a burgeoning field of research that investigates how immune cells harness nutrients to drive their growth and functions. Myeloid cells play a pivotal role in tumor biology, yet their metabolic influence on tumor growth and antitumor immune responses remains inadequately understood. This Review explores the metabolic landscape of tumor-associated macrophages, including the immunoregulatory roles of glucose, fatty acids, glutamine, and arginine, alongside the tools used to perturb their metabolism to promote antitumor immunity. The confounding role of metabolic inhibitors on our interpretation of myeloid metabolic phenotypes will also be discussed. A binary metabolic schema is currently used to describe macrophage immunological phenotypes, characterizing inflammatory M1 phenotypes, as supported by glycolysis, and immunosuppressive M2 phenotypes, as supported by oxidative phosphorylation. However, this classification likely underestimates the variety of states in vivo. Understanding these nuances will be critical when developing interventional metabolic strategies. Future research should focus on refining drug specificity and targeted delivery methods to maximize therapeutic efficacy.

## Therapeutic potential of targeting macrophage metabolism in cancer

Immunometabolism is a rapidly growing area of study that explores how immune cells employ various nutrients to support their growth and functionality. The metabolic programming of immune cells has wide-ranging effects on different disease processes, and a true understanding of these processes is critical to the future of immunotherapies for diseases. In solid tumors, tumor-associated macrophages (TAMs) are abundant and control multiple aspects of tumor growth (1, 2), including immune suppression and evasion through mechanisms such as TGF-β and IL-10 (3–8); the promotion of angiogenesis through the secretion of VEGF (9–12), which mediates resistance to chemotherapy by protecting tumors from oxidative stress; and the promotion of tumor growth after radiation (13–18). Despite their central role in tumor biology, we still lack a proper understanding of how TAM metabolism influences tumor growth and antitumor immune responses. Understanding the specific metabolic functions in the tumor microenvironment (TME) is critical for developing tumor-extrinsic immune-mediated therapies to improve cancer outcomes.

## TAMs coordinate an immunosuppressive TME

**Figure 1:**
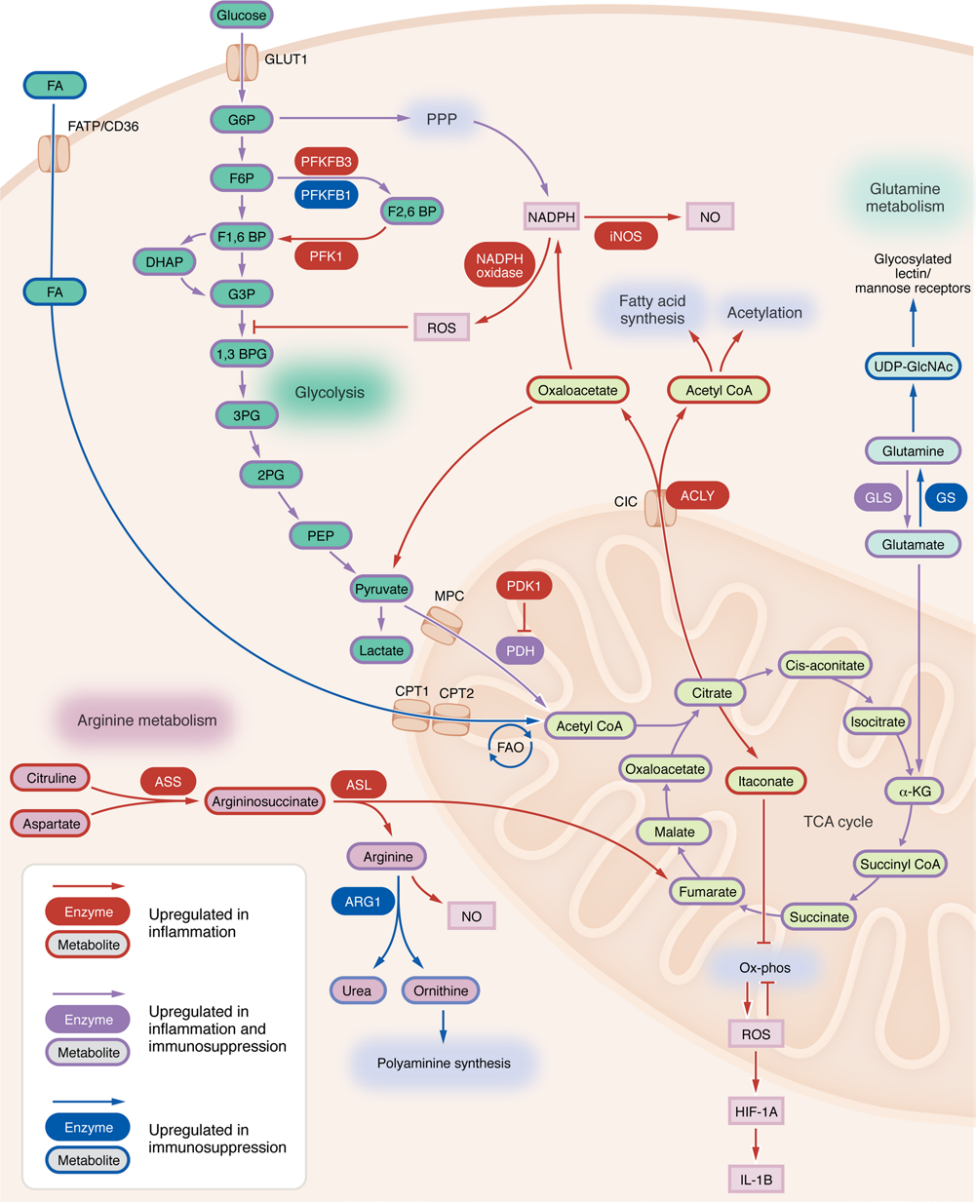
The metabolic landscape of myeloid metabolism and its association with activation status. The diagram depicts the different metabolic pathways in myeloid cells, with red lines indicating a metabolic association with inflammatory phenotypes, and blue lines associated with immunosuppressive or “alternatively” activated macrophages. Several metabolic pathways are associated with both inflammatory and immunosuppressive phenotypes, as denoted by purple lines. BPG, 1,3-bisphosphoglycerate; BP, bisphosphate; CIC, the citrate carrier; FA, fatty acids; G6P, glucose-6-phosphate; F6P, fructose-6-phosphate; F1,6BP, fructose-1,6-bisphosphate; F2,6BP, fructose-2,6-bisphosphate; G3P, glyceraldehyde-3-phosphate; GS, glutamine synthetase; 2PG, 2-phosphoglycerate; 3PG, 3-phosphoglycerate; PEP, phosphoenolpyruvate; MPC, mitochondrial pyruvate carrier; ACLY, ATP-citrate lyase; UDP-GlcNAc, uridine diphosphate N-acetylglucosamine.

Most circulating myeloid cells arise from stem cells in the bone marrow (termed hematopoietic lineage cells), and they include neutrophils, basophils, mast cells, dendritic cells, macrophages, and monocytes. In solid tumors, these immune cells play diverse roles in tumor progression, metastasis, and immunosuppression. In this Review, we will be focusing on TAMs and their immature precursors, monocytes (19). TAMs are distinct from the resident microglia population, which is derived from yolk sac progenitors as opposed to hematopoietic lineages (20). Tissue-resident cells, such as microglia in the brain, Langerhans cells in the skin, and liver-resident Kupffer cells, also play roles in malignancies (21–25), but they will not be discussed in this Review. The immunological status of TAMs can be aligned to proinflammatory M1 or immunosuppressive M2, but this is an oversimplification, and TAM immune phenotypes exist in a continuum. Because of this, there are conflicting results regarding the attribution of one metabolic pathway ascribed to a specific TAM immune phenotype. Notably, there is no direct association of a specific metabolic pathway with a TAM polarization state. As illustrated in Figure 1, we have provided an overview of TAM metabolic processes and their relative contribution to pro- or antiinflammatory phenotypes.

## Aerobic glycolysis versus mitochondrial glucose oxidation in TAMs

Metabolism is required for the proper function of all cells, and it can be categorized into catabolic (from the Greek root of “breaking down”) or anabolic (from the Greek root word of “upward”) metabolism. Most studies on immunometabolism in tumors have focused on catabolic processes, as nutrient scarcity typically drives catabolism in immune cells (26). The term “glycolysis” typically refers to the breakdown of glucose to lactate, even though it truly refers to the generation of pyruvate. The conversion of pyruvate to lactate in the presence of oxygen is defined as “aerobic glycolysis” or the “Warburg effect.” The importation of glucose-derived pyruvate into the mitochondria is called glucose oxidation or aerobic respiration. In this Review, we will use aerobic glycolysis to refer to lactate production and oxidative phosphorylation (OXPHOS) or tricarboxylic acid (TCA) cycle when discussing glucose oxidation.

Analysis of macrophages demonstrates an upregulation and dependence on aerobic glycolysis in response to inflammatory stimuli such as TLRs (27). Activation of TLR4 by LPS or other pathogen-associated molecular patterns results in elevated aerobic glycolysis through the PI3K/AKT signaling cascade; an increase in lactate production; expression of activation surface markers, such as CD40, CD80, and CD86; and elevated GLUT1 glucose transporters — effects that can be reversed in the presence of immunosuppressive IL-10 signaling (27, 28). Subsequent studies have shown that there is a metabolic “break” of glucose oxidation centered within the TCA cycle. This interruption typically occurs at the isocitrate dehydrogenase (IDH) step of the TCA cycle. Specifically, it involves the conversion of isocitrate to α-ketoglutarate (α-KG), a critical point in the cycle in which citrate normally feeds into subsequent oxidative OXPHOS processes. The break, known as “reverse TCA cycle flux” or “TCA cycle rewiring,” involves the diversion of citrate away from the traditional energy production pathway and its conversion into itaconate, a metabolite with immunomodulatory properties. This metabolic rewiring is predominantly observed in macrophages and has been linked to their inflammatory responses. While this metabolic rewiring is prominently recognized in inflammatory macrophages and dendritic cells, further investigation is required to determine whether this phenomenon extends to other cell populations (27, 29, 30).

Inflammatory processes induce a break in the TCA cycle so that intermediates can be used as anabolic intermediates in other pathways that support the inflammatory response (27, 30, 31). Examples of this include (a) the malonylation of glyceraldehyde 3-phosphate dehydrogenase preventing its binding to TNF-α transcripts; (b) the shunting of succinate to stabilize HIF-1α via inhibition of prolyl hydroxylase domain (PHD) enzymes; and (c) the accumulation of citrate and itaconate to inhibit OXPHOS, thereby allowing the macrophages to assume a more glycolytic phenotype (32–35). Succinate is shunted out of the TCA cycle to stabilize HIF-1α, further promoting glycolysis and the production of IL-1β (35). Citrate can be transported out of the mitochondria to be used as an alternate source of cytosolic NADPH via the IDH1 and IDH2 shuttle (36). Indeed, the downstream product of IDH1 and IDH2, α-KG, can shift inflammatory activation by suppressing HIF-1α activity and functioning as a substrate for the demethylation of H3K27 (37, 38). Thus, the ratio of α-KG to succinate correlates with the inflammatory activity of myeloid cells (30, 39). Additionally, decreased rates of OXPHOS are thought to be due to the elevated expression of NOS, a key enzyme producing NO that is capable of reversibly binding complex I in the electron transport chain thereby inhibiting downstream OXPHOS (31, 40, 41). These studies highlight both a reduction in TCA activity and a perturbation in OXPHOS in inflammatory macrophages, as they are both dependent on each other to function.

Notably, this break in the TCA cycle is not as straightforward among all inflammatory processes. For example, mitochondrial OXHPOS is required for activation of the NLRP3 inflammasome (42, 43). Inhibition of mitochondrial electron transport chain complexes I, II, III, and V effectively blocks the activation of the NLRP3 inflammasome. The introduction of exogenous enzymes capable of restoring the function of mitochondria, without inducing the production of ROS, successfully rescues NLRP3 inflammasome activation in the absence of native mitochondrial complex I or complex III activity. In another critical study, authors identified that TCA metabolism is necessary for an inflammatory response (44). In this study, shortly after TLR ligation, macrophages rapidly generate acetyl-CoA from TCA-generated citrate needed to fuel histone acetylation promoting the expression of inflammatory genes.

How can aerobic glycolysis and a broken TCA cycle be critical for inflammatory macrophage activation, while intact TCA metabolism is also essential? To untangle this apparent paradox, one needs to consider the temporal component of these processes. Upon LPS and IFN-γ stimulation, a two-stage remodeling of the TCA cycle occurs: an early stage with a temporary accumulation of intermediates, like succinate and itaconate, and a late stage in which these metabolites diminish, resulting in a progressive breakdown in TCA/OXPHOS, which accompanies inflammatory cell activation (29). When put into a broader context, the early stages of inflammatory macrophage activation require intact TCA/OXPHOS, but this breaks down over time as inflammatory cells become reliant on aerobic glycolysis. Therefore, the simplified notion that aerobic glycolysis is preferential for inflammatory activation comes with the caveat of longitudinal kinetics.

In contrast to inflammatory cells, immunosuppressive myeloid cells have been historically considered less dependent on aerobic glycolysis and more mitochondrial dependent; however, this is an oversimplification. The emergence of pyruvate dehydrogenase kinase 1 (PDK1) has been shown to be a key regulatory step in macrophage polarization, promoting proinflammatory outcomes by restricting commitment to the TCA cycle by inhibition of pyruvate dehydrogenase, whereas loss of PDK1 promotes antiinflammatory outcomes (45). Under metabolite-restricted conditions, the glycolysis inhibitor 2-deoxyglucose (2-DG) has been used to demonstrate that glycolysis is necessary to fuel immunosuppressive myeloid functions, further contributing to the complexity of metabolic requirements for immunosuppressive myeloid cells in tumors. More specifically, 2-DG can inhibit TAM polarization after immunosuppressive IL-4 treatment but only in glucose-limited conditions and not in conditions sufficient to maintain TCA cycle function, such as high galactose or glutamine supplementation. These data suggest that immunosuppressive TAMs prefer glucose as a fuel for TCA cycle support but have the means necessary to maintain TCA function in nutrient-restricted conditions such as glycolysis inhibition (46, 47). Reinforcing these findings, a seminal study identified that mTORC2 and IRF4 work in parallel to upregulate glucose metabolism and promote immunosuppression in tumors (48). Based on these findings, it is evident that immunosuppressive TAMs prefer to use glucose metabolism to fuel TCA cycle turnover and thereby promote OXPHOS, but they are also capable of adapting their metabolic programing to utilize other sources.

Research by the Reinfeld group serves as an example of the highly glycolytic nature of TAMs. Their work showed that in colorectal cancer TAMs consume more glucose than any other cells within the TME, surpassing even the tumor cells (49). This study indicated that glutamine is the preferred substrate for tumors in vivo, calling into question how much the Warburg effect is essential to tumor growth in vivo. Indeed, two landmark studies have shown the necessity of OXPHOS in tumor growth (50–53). Collectively, the findings from these studies indicate that, in an in vivo setting, the target of glycolytic inhibition may be primarily the TAMs rather than tumor cells.

## The pentose phosphate pathway supports inflammatory myeloid activation

The pentose phosphate pathway (PPP) is critically associated with glycolytic metabolism, but it is understudied due to a lack of pathway-specific reagents. The PPP is an anabolic pathway that shares several enzymes with the glycolytic pathway and is necessary for shunting glucose-6-phosphate away from glycolysis to rapidly restore the depleting NADPH pool in inflamed myeloid cells (30). Although inflamed myeloid cell metabolism relies on PPP replenishment of NADPH, immunosuppressive TAMs upregulate the sedoheptulose kinase CARKL, suppressing flux to the PPP (54). NADPH, in this context, serves as a pool of reductive power to replenish rapidly depleting glutathione pools, thus protecting the cell from ROS. Myeloid cell reliance on PPP to produce NADPH further illustrates the complex coordination of metabolic intermediates in myeloid cell inflammation.

## Fatty acid metabolism

Fatty acid oxidation (FAO) is another essential input into the mitochondria and a robust source of metabolic acetyl CoA, FADH_2_, and NADH when glycolysis is insufficient for the energetic needs of a cell. Fatty acids can be imported via CD36 and FATP1 or generated de novo via lipolysis (55, 56). FAO occurs after fatty acids are transported through the mitochondrial membrane via the carnitine palmitoyltransferase (CPT) system (57–59). CPT1 is located on the outer mitochondrial membrane and transports long-chain fatty acids but not medium- or short-chain fatty acids (60). CPT1 converts acyl-CoA to acyl-carnitine, which then can be converted to acyl-CoA by inner mitochondrial membrane-bound CPT2. It was historically thought that immunosuppressive myeloid cells increase their reliance on FAO to maintain their high energetic needs; however, this interpretation assumed that etomoxir was an FAO inhibitor. The studies that demonstrated that FAO is important for immunosuppressive myeloid function used etomoxir at concentrations that inhibited mitochondrial metabolism, resulting in changes to acetyl-CoA pools in macrophages or induced severe oxidative stress (61, 62). Additionally, in a subsequent study, CPT2 knockout in bone marrow–derived macrophages did not increase oxygen consumption rates in response to the addition of palmitate in the presence of IL-4 while maintaining immunosuppressive gene expression (*Arg1*, *Mgl2*, *Retnla*) and immunosuppressive markers (CD206 and CD301), demonstrating that FAO, as assessed by oxygen consumption rates, is not required for IL-4–induced immunosuppressive polarization (63). Another study using concentrations of etomoxir that have more selective effects demonstrated that FAO is dispensable for M2 polarization (64). As such, the role of FAO on myeloid immunosuppression may be more dispensable than previously thought.

Bone marrow–derived myeloid cells activated with LPS tend to increase fatty acid anabolism, in contrast to the fatty acid catabolism increase in immunosuppressive myeloid cells. Increased fatty acid uptake and de novo synthesis are hallmarks of LPS stimulation and revolve around SREBP1 signaling in late-phase activation of inflammatory myeloid cells (65, 66). TLR4 signaling, which upregulates aerobic glycolysis, is dependent on the enzyme fatty acid synthase to generate palmitate and is attenuated when fatty acid synthase is inhibited (67). LPS-mediated increases in fatty acid synthesis result in accumulation of lipid droplets; increased expression of PGE2, IL1-β, and NOS; and the resulting production of NO, while reducing the ability of the TCA cycle to perform FAO (68–70). Further complicating the paradigm, studies with etomoxir in LPS-stimulated myeloid cells have shown an attenuation of inflammatory outputs, specifically mitochondrial ROS and NLRP3 inflammasome pathways, which could be attributed unknown off-target effects of the drug or an unknown dependence on some level of FAO by inflammatory myeloid cells (71, 72).

## Targeting glycolysis and mitochondrial metabolism in TAMs

Given the pivotal influence of glucose metabolism on TAM functions, reconfiguring these metabolic processes may offer a promising pathway to enhance current immunotherapy treatments. When considering combinatorial approaches to treating cancer, the metabolic phenotype and tumor-associated immune cell compartment should be considered. A general trend across most immunologically “cold” tumors is a hypoxic and metabolite-scarce TME due to resource limitations, leading to an exclusion of cytolytic CD8^+^ T cells and promotion of the activity of immunosuppressive TAMs (73). Thus, restricting the ability of TAMs to support this phenotype via metabolic inhibition may be a novel approach to controlling the TME, with the added benefit of also inhibiting tumor cell growth directly, as tumor cells tend to prefer high metabolic rates that rely on increased glucose consumption, increased glycolysis, and elevated TCA cycle turnover.

Metformin, an antidiabetic drug that has numerous off-target effects, including inhibiting mitochondrial complex 1, has been used to target TAM metabolism by modulating the AMPK/mTOR/NF-κB signaling axis (74–78). These studies indicate that metformin can promote the antitumor phenotype of TAMs. However, the specific target of metformin that is responsible for proinflammatory skewing is not well defined.

2-DG is another metabolic inhibitor used in tumor treatment with effects that can be partially attributed to TAM reprogramming. In preclinical models of melanoma, 2-DG decreases TAM expression of *Arg1*, *Fizz*, *Mrc1* (encoding CD206), and *Vegf*, indicating that 2-DG diminishes immunosuppression within the TME (46). In another study, conditioned media from pancreatic tumor cells increased the aerobic glycolysis in macrophages, which resulted in prometastatic/angiogenic phenotypes, whereas treatment with 2-DG could reverse these effects (79). Such evidence further suggests that TAMs can rely on glucose metabolism to support both protumor and antiinflammatory phenotypes in vivo. Notably, 2-DG treatment has several off-target effects (80, 81). Therefore, caution should be used when interpreting the metabolic effects of these compounds on TAM activities in tumors.

Phosphofructokinase (PFK) inhibition has also been shown to abrogate inflammatory phenotypes in macrophage cultures in vitro. However, tumor-conditioned media is also capable of inducing expression of PFK in immunosuppressive TAMs (82). Pyruvate kinase M2 (PKM2) exerts control over TAM expression of PD-L1 in pancreatic cancer, while also supporting tumor cell growth, exemplifying the dual benefit of glycolytic perturbation (83, 84). Indeed, lactylation of PKM2 has been shown to prevent inflammatory activation of macrophages (85). An inhibitor of HIF-1α, PX-478, halts glycolysis in tumor cells, leading to better responses to radiotherapy in prostate cancer, prevention of metastases in small cell lung cancer, and enhanced immunogenic cell death in pancreatic cancer (86–88). Inhibition of HIF-1α also triggers a shift from immunosuppressive phenotypes and decreased PD1/PD-L1 expression (89, 90). Therefore, targeting glycolysis (and FAO) may exert therapeutic impact on immunosuppressive TAMs (91).

**Figure 2:**
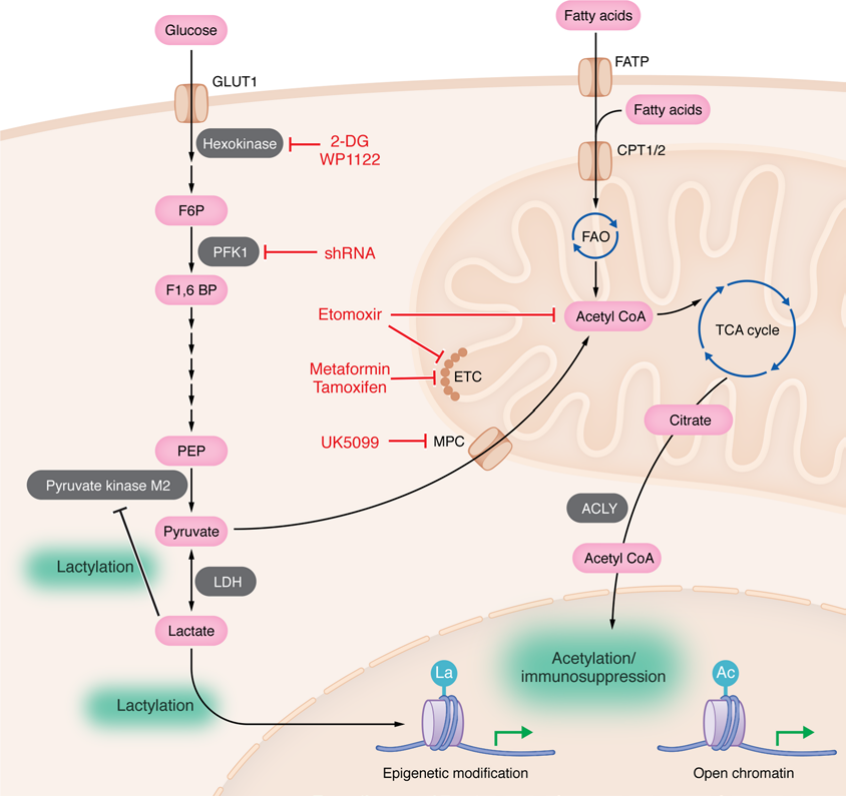
Glycolysis, oxidative phosphorylation, and fatty acid oxidation in immunosuppressive myeloid cells and therapeutic strategies. 2-Deoxyglucose (2-DG) and the BBB permeable prodrug (WP1122) are glucose analogs that have been shown to prevent tumor growth and perturb TAM. shRNA targeting phosphofructokinase 1 (PFK1) has been shown to prevent immunosuppressive macrophages. UK5099, inhibits the mitochondrial pyruvate carrier (MPC) and can prevent immunosuppressive macrophage phenotypes induced by lactate metabolism in TAM. Etomoxir, while historically thought to be a fatty acid oxidation inhibitor (FAO), has off-target effects responsible for inhibiting alternative activation. Metformin, which potently inhibits immunosuppressive macrophage activation, while originally thought to be an AMPK activator, can also inhibit complex 1 of the electron transport chain (ETC). Tamoxifen has shown anti-ETC activities that may underlie its suppression of immunosuppressive macrophage activation. ACLY, ATP-citrate lyase.

For many brain tumor malignancies, a translational barrier for metabolic manipulation is sufficient blood-brain barrier (BBB) penetration. There are a variety of strategies for overcoming the BBB that do not rely on medicinal chemistry strategies, such as BBB-opening ultrasound or conjugating long-carbon chains to targeting RNA linkers, but these have not been leveraged yet for metabolic therapeutic approaches (92–95). CNS penetrant drugs, while scarce, have been developed, such as the 2-DG mimetic dichloroacetate (96). The 2-DG prodrug WP1122 has direct cytotoxic effects on glioblastoma cell survival when combined with histone deacetylase inhibitors (97). Dichloroacetate is being evaluated in an ongoing phase II clinical trial of patients with glioblastoma (NCT05120284) (91). Another metabolic modulation strategy that is in clinical trials is tamoxifen, which is primarily used as an estrogen receptor mimetic but also has electron transport chain inhibitory properties (98–100). An ongoing phase II clinical trial using tamoxifen has demonstrated safety (NCT04765098), but results of efficacy have not yet to be shown. In glioblastoma, nutrient limitations result in the glioma cells relying on FAO to support cellular processes and proliferation, which can be targeted with the use of etomoxir (101, 102). These findings in glioblastoma need to be carefully interpreted, as the heterogeneity of the tumor and TME, along with off-target effects, may lead to an overinterpretation of the reliance of tumor cells on fatty acid metabolism (103). Further work to produce brain-penetrant glycolysis– and OXPHOS–modulating drugs is needed to adequately target the metabolic phenotypes of TAMs within the TME. An overview of the strategies used to perturb glycolysis, oxidative phosphorylation, and fatty acid oxidation in TAMs can be found in Figure 2.

## Arginine metabolism

The amino acid arginine provides the most direct link between metabolites and myeloid functions in tumors. Arginine is an essential nutrient in tissues, serving as a key component in protein synthesis. Studies in pancreatic cancer identify it as the most depleted metabolite in the interstitial milieu (pyruvate, tryptophan, and cysteine were also significantly depleted) (104). Our own data show similar depletion in glioblastoma, along with several other limiting amino acids, such as glutamine and l-aspartic acid, which is likely secondary to consumption by myeloid cells (105, 106). Arginine is by no means the only metabolite depleted in tumors, as other studies have shown that serine, exogenous fatty acids, aspartate, glutamine, and serine can also be limiting (107–110).

As tumors gain biomass, there is a requirement for arginine as a proteinogenic amino acid (111). Arginine metabolism has been at the center of macrophage/myeloid cell biology for over two decades. The original M1/M2 description of macrophage polarization hinges largely on the split between metabolic selection of iNOS activity versus arginase-1 (Arg1) activity, respectively (112). While it is recognized that strict M1/M2 phenotypes are an oversimplification in vivo, it holds true that myeloid cells consume large amounts of arginine (113). This myeloid cell consumption of arginine outcompetes T cells, which requires this metabolite for T cell proliferation (114). Arginine deprivation perturbs T cell activation, and L-arginine supplementation enhances T cell survival and effector functions in vivo (114). This consumption also has effects on tumor cells. A recent study showed that pancreatic tumors must upregulate arginine biosynthesis to compensate for this competition for local arginine (115).

The byproducts of arginine metabolism, polyamines, have long been associated with both increased malignancy and immunosuppression (116). In the context of glioblastoma, previous work from our group showed that TAMs actively produce polyamines to buffer themselves within the acidic TME (117). We also found that blockade of polyamine synthesis was sufficient to enhance survival in preclinical models of glioblastoma, an effect dependent on host immunity. Our group has recently found that TAMs produce creatine from arginine, which is being fed to tumor cells to allow their survival in the hypoxic niche (118). Arginine is also a substrate for proline, which promotes the production of collagen fibrils and subsequent fibrosis, which promotes immune exclusion from tumors (119–121). Finally, there is evidence that polyamines can directly promote macrophage alternative activation. The hypusination of EIF5a, facilitated by the polyamine spermidine, exerts direct regulation over the transcription and translation of mitochondrial proteins in macrophages (122). The polyamines generated by IL-4–induced macrophages promote alternatively activated macrophage activation by stimulating OXPHOS and the TCA cycle. Other byproducts of arginine metabolism produced by myeloid-derived suppressor cells (MDSCs) or macrophages, such as NOs and peroxynitrites, directly inhibit several aspects of T cell activity and function through nitrosylation (123, 124).

iNOS/NOS2 is an enzyme that is expressed in cells in response to inflammatory activity or hypoxia. It produces NO by converting l-arginine to l-citrulline (125, 126). NO reprograms macrophage mitochondrial metabolism by generating nitroxyl, which inhibits the pyruvate dehydrogenase complex. This causes macrophages to enter a glycolytic state, thereby reducing ATP production by the TCA cycle (127–129). These changes, which are mediated by LPS, promote inflammatory polarization, which leads to increased cytokine and NO production by macrophages (128). This process is kept in balance by the immunosuppressive cytokine IL-10, which is modulated by a commitment to aerobic glycolysis and, in turn, modulates NO-mediated suppression of OXPHOS (130). Proinflammatory macrophage polarization can have a variety of pro-oncogenic effects, including promoting pathways for angiogenesis, the epithelial-mesenchymal transition, cancer cell survival, proliferation, and metastasis (125, 126). iNOS/NOS2-targeting therapies have been studied in several in vitro and in vivo models (131–133). However, clinical trials of NOS inhibitors are scarce because of concerns about off-target effects (126).

## Therapeutic targeting of arginine metabolism

**Figure 3:**
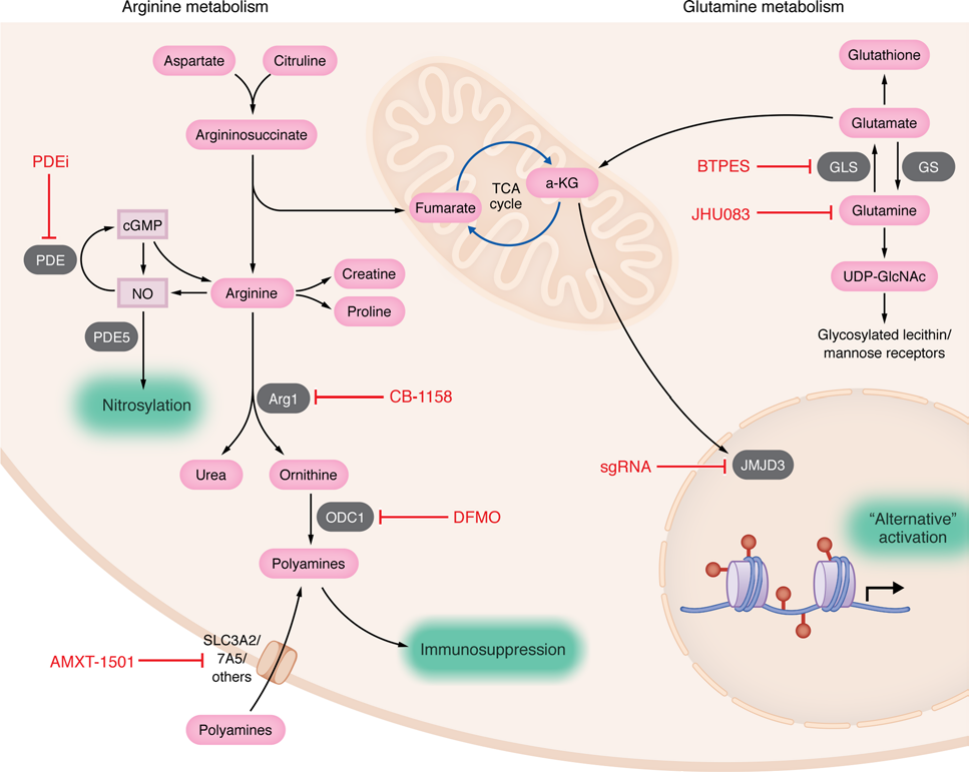
Arginine and glutamine metabolism in immunosuppressive myeloid cells and therapeutics. Arginine metabolic processes and therapeutic strategies are shown on the left. Phosphodiesterase inhibitors (PDEi) have been used both preclinically and clinically to inhibit MDSC/TAM functions in several tumor models. The highly specific inhibitor of arginase 1, CB-1158, blocks TAM function and promotes antitumor immunity. Monotherapeutic difluoromethylornithine (DFMO) alone or in combination with the polyamine uptake inhibitor AMXT1501 can promote T cell infiltration into tumors and promote immunotherapeutic efficacy. The role of glutamine metabolism in immunosuppressive macrophage activation and therapeutic strategies are shown on the right. BTPES (Bis-2-(5-phenylacetamido-1,3,4-thiadiazol-2-yl)ethyl sulfide) and JHU083 ((Ethyl 2-(2-Amino-4-methylpentanamido)-DON) are inhibitors of glutaminase 1 (GLS) and can prevent alternative macrophage activation. (We note, however, that JHU083 is a nonselective inhibitor that inhibits all glutamine-utilizing enzymes.) sgRNA ablation of Jumonji domain-containing protein-3 (JMJD3) can also blunt glutamine-induced alternative activation of macrophages.

Due to the apparent central importance of arginine catabolism in promoting immunosuppression, several strategies have been developed to target this pathway to promote antitumor immunity (Figure 3). The small-molecular inhibitor CB-1158 that blocks Arg1 enhances immunotherapy in murine models of colorectal tumors, melanoma, and breast cancer and has now been advanced to clinical trials (134). Although it was demonstrated safe in the context of immunotherapy, efficacy studies have not yet been reported (135). Several other Arg1 inhibitors are being tested or are in development for future therapeutic use (136, 137).

The inhibition of polyamines is another strategy for modulating arginine metabolism. Several studies have shown that polyamine inhibition using difluoromethylornithine (DFMO) can be an effective way to enhance immunotherapy (138–141). In an initial study, combination treatment involving DFMO and a synthesis inhibitor (AMXT-1501) effectively reversed immune suppression in melanoma mouse models (142). Subsequently, in a preclinical model of melanoma and colon carcinoma, inhibition of polyamine activity led to increased tumor infiltration of granzyme B^+^ IFN-γ^+^ CD8^+^ T cells, while concurrently reducing immunosuppressive TAMs (143). These findings align with those of our recent investigation, wherein we observed that polyamine blockade facilitated the infiltration of CD8^+^ T cells and synergistically enhanced animal survival when combined with anti-PD1 or anti–PD-L1 immunotherapy in murine models of glioblastoma (117). Furthermore, the combination of polyamine blockade and immune checkpoint blockade exhibited a synergistic effect in murine models of mammary carcinogenesis (144). Collectively, these studies demonstrate that polyamine blockade has the potential to modify the TME in a manner that promotes T cell–dependent antitumor responses.

Finally, blocking iNOS-mediated immunosuppression has also shown effects in promoting an antitumor response. For example, phosphodiesterase-5 (PDE5) inhibitors, such as sildenafil, which interfere in cGMP-dependent iNOS signaling, have been shown to prevent MDSC-mediated immunosuppression (145–147). PDE5 inhibition can reduce iNOS and Arg1 activity in MDSCs, thereby triggering antitumor response and T cell infiltration in preclinical models of colon cancer and breast cancer (145) or in patients with end-stage multiple myeloma (146). PDE5 inhibition also prevents MDSC-induced NK suppression, increasing NK cytotoxicity in murine models and in humans with abdominal malignancy (147). A phase II study has been initiated to investigate the effects of combining nivolumab, tadalafil, and oral vancomycin in patients diagnosed with refractory primary hepatocellular carcinoma or liver-dominant metastatic cancer originating from colorectal or pancreatic cancers (148). The results of this combinatorial therapy have not yet been reported.

## Other amino acid oxidizing pathways in TAMs

TAMs also express other immunosuppressive amino acid–oxidizing enzymes, such as IDO and IL4i1. IDO is an intracellular, tryptophan-metabolizing enzyme that functions through its catalysis of the rate-limiting step of the kynurenine pathway. IDO is highly expressed in TAMs and MDSCs in the TME and plays a role in tumor immune escape (149, 150). Tryptophan metabolism and subsequent depletion via IDO expression in macrophages has been shown to inhibit antigen-specific T cell proliferation and activation (151). Additionally, kynurenine has been shown to directly activate the aryl hydrocarbon receptor, and this activation leads to the generation of immunosuppressive FoxP3^+^ regulatory T cells (152). In patients with breast cancer, increased IDO-expressing MDSC populations are correlated with increased amounts of these FoxP3^+^ regulatory T cells and a poor prognosis (153). Several IDO inhibitors in combination with other immune therapeutics are being actively investigated in a number of clinical trials across a broad array of cancers.

IL4i1 is a l-amino acid oxidase secreted by APCs that primarily functions to oxidize phenylalanine (154). Expression of IL4i1 in TAMs has been shown to prevent T cell proliferation and cytokine production, decrease the CD8^+^ T cell response, and promote the differentiation of naive CD4^+^ T cells into FoxP3^+^ regulatory T cells (155, 156). In addition to suppressing the antitumoral T cell response, IL4iL expression has also been shown to recruit immunosuppressive MDSC populations to the TME in a mouse model of melanoma (157).

## Glutamine metabolism

Glutamine is the most abundant amino acid found in the human body, and it is conditionally essential in times of catabolic stress. Once transported into a cell, glutamine is lysed into ammonium ion and glutamate by mitochondrial glutaminases in a process known as glutaminolysis. The resultant glutamate can subsequently be converted into α-KG by glutamate dehydrogenas or aminotransferases, allowing anapleurotic reactions to support TCA function. As a major source of carbon and nitrogen, glutamine is essential for production of amino acids, purine, pyrimidines, and lipids. Additionally, glutamine-derived glutamate can be utilized in synthesis of glutathione, which is used to neutralize ROS and maintain redox balance. As highly proliferative cells with large anabolic requirements, tumor cells exhibit particularly high levels of glutamine uptake and dependence.

Glutamine metabolism plays a pivotal role in macrophage activation and polarization. Glutamine-derived α-KG is required for the differentiation of macrophages to an antiinflammatory, immunosuppressive phenotype, and induction of endotoxin tolerance (30). Glutamine deprivation and glutaminolysis inhibition using a GLS1 inhibitor decreased expression of immunosuppressive genes while upregulating inflammatory genes such as *IL-1*β and *Tnf* in bone marrow–derived macrophages (37). The α-KG derived from glutamine limits the activation of an inflammatory macrophage phenotype via suppression of the NF-κB pathway by functioning as a substrate for PHD inhibition of HIF-1α and suppression of IKKβ (37). Furthermore, α-KG directly supports epigenetic changes, specifically JMJD3-dependent H3k27me3 demethylation, allowing for the transcription of key immunosuppressive genes like *Il10*, *Tgfb*, and *Arg1*, and it can reverse chronic inflammatory phenotypes in alveolar macrophages (37, 38). It is important to note that while α-KG can be produced by IDH2/3 within the TCA cycle, or IDH1 within the cytosol, prior studies have exclusively focused on α-KG derived from glutaminolysis (37–39). Extracellular glutamine, along with arginine, is required to produce NO in murine macrophages. Thus, glutamine contributes to the polarization of macrophages toward an immunosuppressive phenotype like those of TAMs in the TME.

Despite the pivotal role for glutamine metabolism in macrophage polarization and function, very little is known about how these processes work within the TME. Glutamine metabolism is a promising target to sensitize tumors and their immunosuppressive microenvironments toward immunotherapy. JHU083, a prodrug version of the glutamine antagonist 6-diazo-5-oxo-L-norleucine, is a glutamine metabolism inhibitor that is selectively activated in the TME to mitigate toxicity; it has been shown to inhibit tumor growth and promote survival in tumor-bearing mice, particularly in combination with immunotherapy (158). Administration of this prodrug augmented endogenous antitumor immunity, as it promoted activation, proliferation, and memory in tumor-infiltrating lymphocytes (158). Additionally, JHU083 inhibits the recruitment of immunosuppressive MDSCs to the TME and induces MDSC apoptosis while simultaneously reprogramming MDSCs and TAMs to a proinflammatory antitumor phenotype (159). Notably, glutamine inhibition via JHU083 increased the effectiveness of anti-PD1 and anti-CTLA4 checkpoint blockade in tumors that did not benefit from monotherapy (159). An outline of the glutamine metabolic pathway and current targeting strategies can be found in Figure 3.

## Conclusions and future directions

The analysis in this Review indicates that metabolic reprograming is a reasonable strategy for enhancing antitumor immunity in TME. In a general sense, characterization of the immune component of the TME could be considered when deciding the metabolic approaches to alter the TME and could inform an appropriate strategy to directly manipulate local inflammation signals to achieve better outcomes. For example, the TME of glioblastoma is largely myeloid in nature and lacks infiltrating CD8^+^ T cells; thus, targeting myeloid metabolism to shift the TME to a more inflamed phenotype could unlock the therapeutic benefits of anti-PD1 or other checkpoint blockades. Additionally, these metabolic pathways may overlap, likely due to the nutrient-scarce TME; therefore targeting one metabolic pathway may also inhibit tumor cell activity while abrogating immunosuppressive myeloid cell functions. However, current drugs are lacking specificity, such as those with myriad off-target effects (metformin, etomoxir, and others); lacking tumor specificity; or, in the case of CNS tumors, lacking BBB penetrance. These drug design limitations in the metabolic field make them unusable in a precision medicine setting and more so in most brain tumor settings. In general, careful consideration of the location of the tumor, its metabolic niche, and immune cell component are all factors to consider when studying and establishing novel metabolic targets to improve host-mediated immune rejection of the tumor. One strategy would be the use of myeloid cell–targeting lipid-coated nanoparticles (77, 160).

In this Review, we described numerous instances in which metabolic modulation led to improved outcomes both in mono- and combinatorial therapeutic settings. Of the described metabolic pathways within TAMs, arginine metabolism can be prioritized as a pathway for disruption, especially when paired with an immune checkpoint blockade or other immune-based therapy. However, the timing and targeting for this approach would need to be carefully considered, because limiting arginine metabolism within T cells would inhibit their cytotoxic functions and induce cell cycle arrest (114, 161). Aside from arginine metabolic inhibitors, glutaminase inhibitors have the potential to target both immunosuppressive TAMs and the tumor, as glutaminolysis is essential for both cells. Glycolytic inhibitors are also a therapeutic candidate given their effect on TAMs and direct effect on tumor growth.

Overcoming the protumoral role of TAMs, while sparing adaptive immunity, is a significant challenge that remains to be overcome in tumors, and with properly designed drug delivery methods, such as the packaging of therapies within TAM-targeting nanoparticles, proper cell-level specificity may be achieved (77, 162, 163). To highlight the challenges of using therapies to target immunometabolism in tumors we have included tables to illustrate key considerations (Table 1) and other prerequisite for metabolic drug discovery (Table 2) that are essential when considering these approaches. Once these goals are achieved, a new era of precision medicine may include metabolic phenotyping of the patient tumors for targeted metabolomic strategies.

## Figures and Tables

**Table 1:**
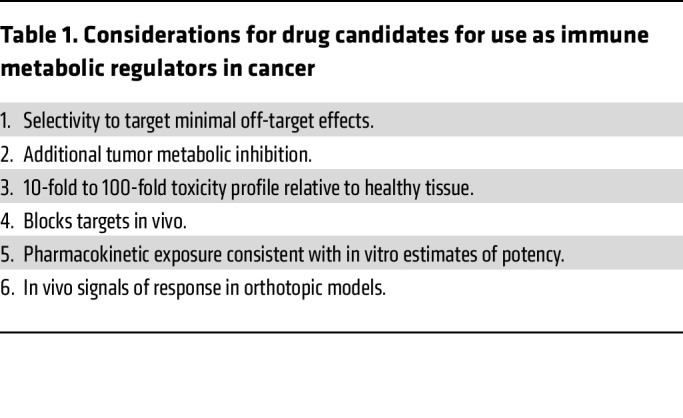
Considerations for drug candidates for use as immune metabolic regulators in cancer

**Table 2:**
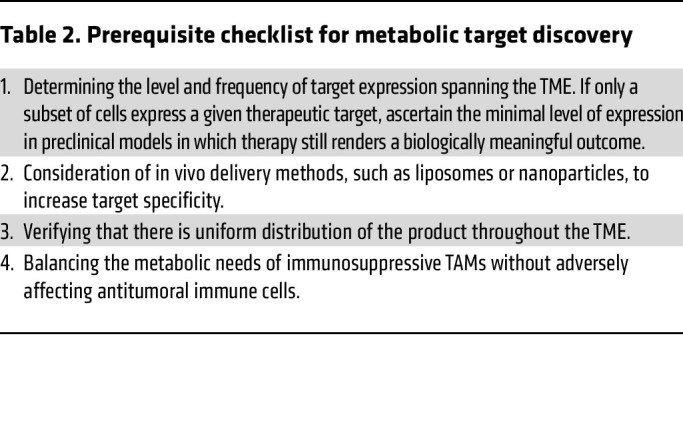
Prerequisite checklist for metabolic target discovery

## Version 1 01/16/2024

Electronic publication
